# Intraductal dissemination of ampullary carcinoma after pancreatoduodenectomy

**DOI:** 10.1186/s40792-019-0740-4

**Published:** 2019-11-08

**Authors:** Koichi Mohri, Kazuhiro Hiramatsu, Yoshihisa Shibata, Motoi Yoshihara, Taro Aoba, Atsuki Arimoto, Akira Ito, Takehito Kato

**Affiliations:** Department of General Surgery, Toyohashi Municipal Hospital, 50 Hachiken-nishi, Aotake-cho, Toyohashi, Aichi 441-8570 Japan

**Keywords:** Ampullary carcinoma, Adenocarcinoma, Pancreatectomy, Intraductal dissemination

## Abstract

**Background:**

Clinical evidence of intraductal dissemination through the pancreatic duct has been rare. We herein describe a case of ampullary carcinoma that disseminated in the remnant pancreas through the pancreatic duct.

**Case presentation:**

A 68-year-old woman underwent SSPPD for ampullary carcinoma. The tumor was diagnosed as adenocarcinoma without lymph node metastasis (T2N0M0, stage IB). Computed tomography (CT) performed 3 years later revealed a 14-mm tumor near the site of the pancreaticojejunal anastomosis. Endoscopic ultrasound-guided fine needle aspiration showed adenocarcinoma that was morphologically similar to the specimen from the first surgery. We diagnosed recurrence of ampullary carcinoma in the remnant pancreas. A total remnant pancreatectomy was performed. We found a white solid tumor at the 20-mm distal side of pancreaticojejunal anastomosis. The tumor was morphologically similar and immunostaining showed a pattern identical to that of the original tumor, suggesting that the two tumors were of the same origin.

**Conclusion:**

The recurrent lesion was most likely the result of tumor cells leaving the tumor and implanting in the remnant pancreatic duct epithelium. Intraductal dissemination of adenocarcinoma is thought to be a cause of remnant recurrence after SSPPD in cases of obstruction of the pancreatic duct or an iatrogenic procedure.

## Background

Intraductal dissemination through the pancreatic duct is a unique mechanism of dissemination. Clinical evidence of this form of dissemination is scant, described only in case reports. Here, we describe a case of ampullary carcinoma that disseminated and implanted in the remaining pancreas through the pancreatic duct after subtotal stomach preserving pancreatoduodenectomy (SSPPD).

## Case presentation

A 68-year-old woman was referred to our hospital for further examination of jaundice. She had no remarkable past history.

Laboratory findings were notable for carcinoembryonic antigen (CEA) level within normal limits; however, carbohydrate antigen 19-9 (CA19-9) level was slightly high (65.9 U/mL), and liver enzyme levels were elevated: aspartate aminotransferase, 155 IU/L; alanine aminotransferase, 98 IU/L; γ-glutamyl transpeptidase, 1620 IU/L; alkaline phosphatase, 1980 IU/L; and total-bilirubin, 5.3 mg/dL.

**Fig. 1:**
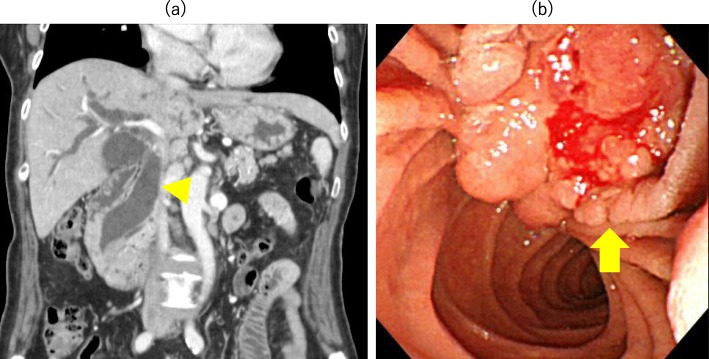
**a** Abdominal CT revealing a dilation of both the common bile duct (arrow head) and the main pancreatic duct. **b** Upper gastrointestinal endoscopy revealing a mass lesion (arrow) in the ampulla of Vater

**Fig. 2:**
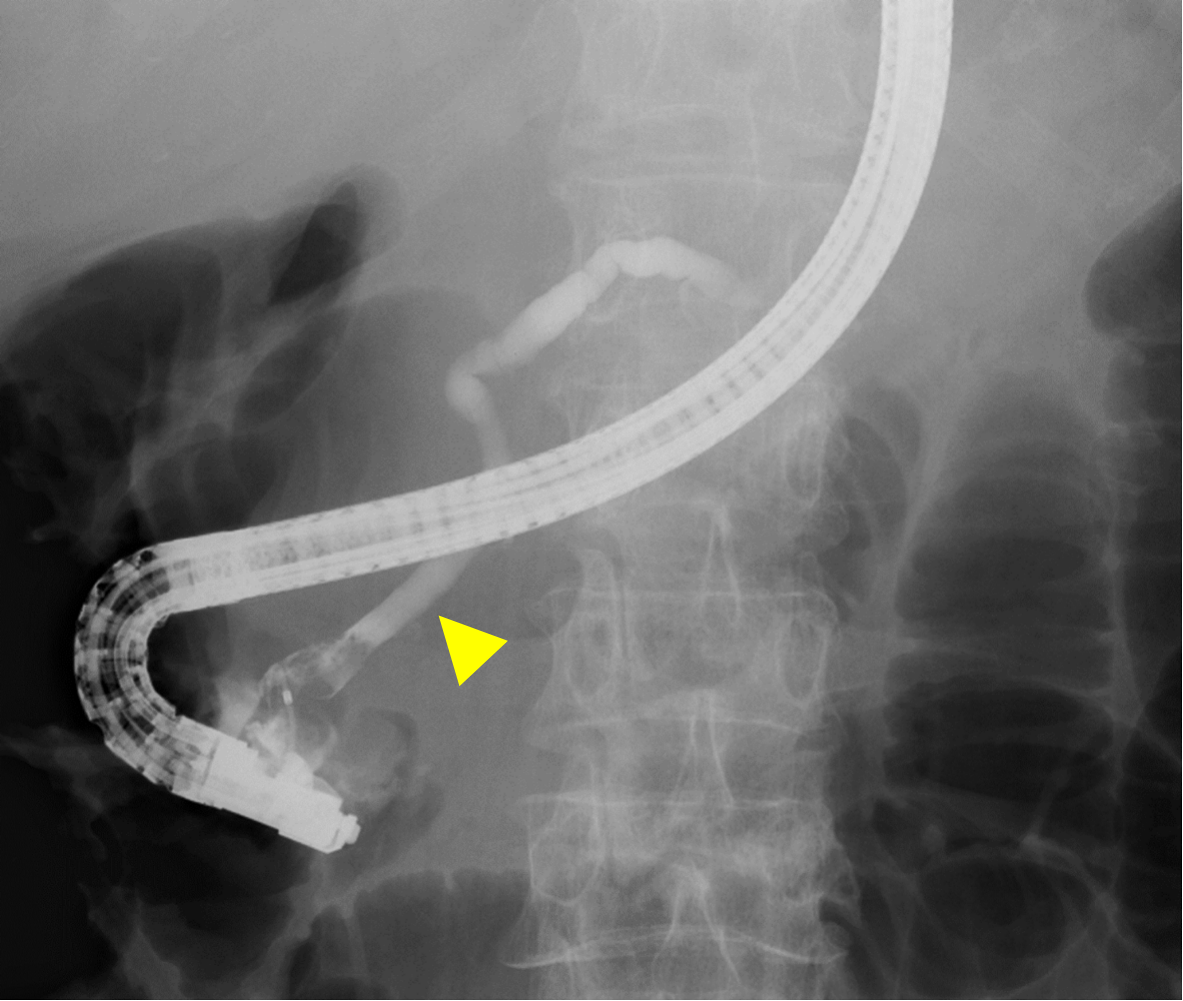
ERCP revealing a dialated main pancreatic duct (arrow head)

Contrast-enhanced CT revealed a 27 × 23-mm irregular mass with a low-contrast effect in the duodenum and dilatation of both the upstream main pancreatic duct and the common bile duct (Fig. 1a). Duodenoscopy revealed a swollen villous tumor with radiating folds at the duodenal papilla (Fig. 1b). Endoscopic biopsy revealed a moderately differentiated adenocarcinoma. Endoscopic retrograde cholangiopancreatography (ERCP) revealed a dilated main pancreatic duct without stenosis (Fig. 2). A biliary stent was inserted endoscopically.

The tumor was diagnosed as an ampullary carcinoma, and SSPPD with modified Child reconstruction was performed. The operating time was 353 min, and total blood loss was 195 mL.

**Fig. 3:**
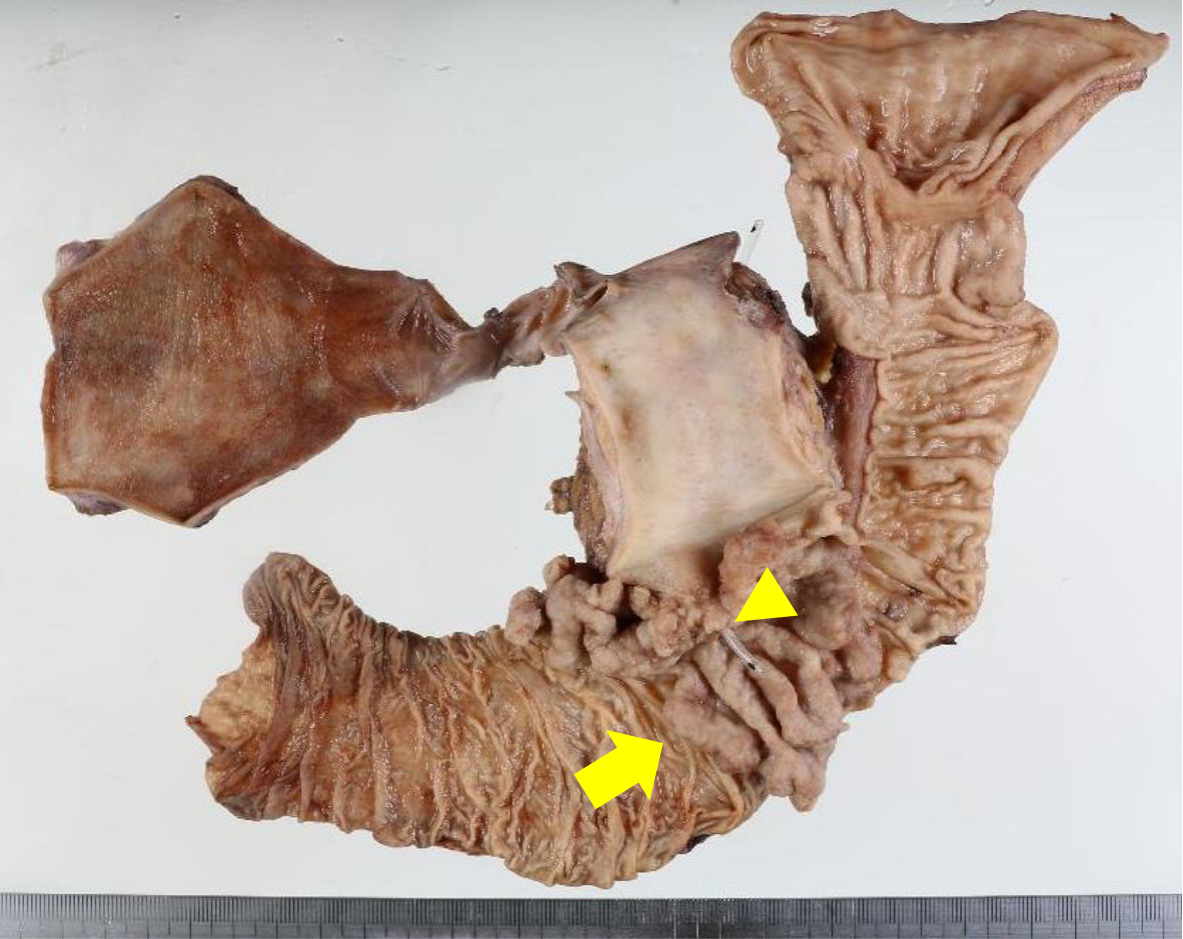
The resected specimen was a large villous tumor measuring 80 × 55 mm (arrow), arising around the orifice of the duodenal papilla (arrow head)

Macroscopically, the tumor measured 80 × 55 mm with a villous appearance around the orifice of the duodenal papilla (Fig. 3). Microscopically, the tumor invaded the sphincter of Oddi and duodenum but not the pancreatic stroma. All surgical margins were negative. The pathological stage was T2N0M0 stage IB. Microscopically, neither invasion of lymph vessels nor microvessels was found. The results of immunohistochemical staining were as follows: cytokeratin7 (−), cytokeratin20 (+), MUC1 (+), and MUC2 (+) (Fig. 8).

**Fig. 4:**
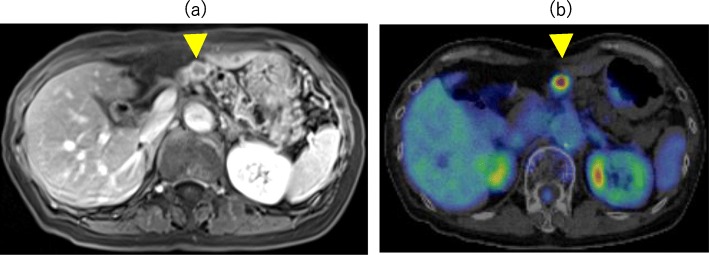
**a** Contrast enhanced MRI. revealing a peripherally-contrasted irregular tumor in the remnant pancreas (arrow head). **b** PET-CT. The maximal standardized uptake value (SUV max) was 6.8 at the tumor (arrow head)

**Fig. 5:**
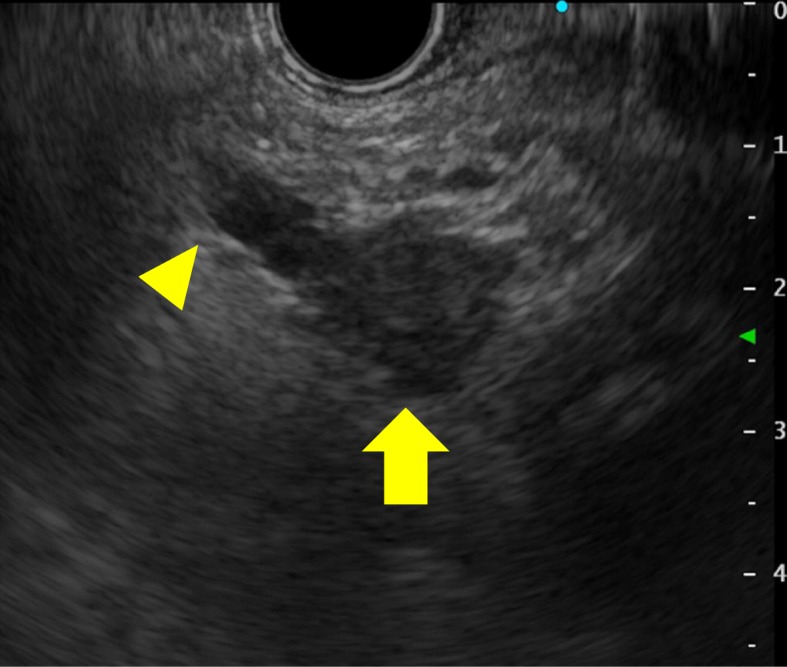
EUS revealing 16-mm hypoechoic tumor (arrow) with irregular margin. There was intraluminal growth without stricture of pancreatic duct (arrow head)

After surgery, the patient was followed up every 6 months. Three years later, magnetic resonance imaging (MRI) revealed a peripherally contrasted 14-mm-diameter region near the pancreaticojejunal anastomosis (Fig. 4a). Laboratory data including CEA and CA 19-9 were within normal limits. Positron emission tomography/computed tomography (PET-CT) revealed an accumulation of SUV max 6.8 at the tumor, while any findings of other metastases were not observed (Fig. 4b). EUS revealed 16-mm hypoechoic tumor with irregular margins. The tumor appeared to originate from the mucosal layer of the main pancreatic duct, subsequently invading surrounding stroma of the pancreas (Fig. 5). EUS-guided fine needle aspiration revealed ampullary carcinoma that was morphologically similar to the specimen from the first surgery. The tumor was diagnosed as recurrence of ampullary carcinoma rather than a new primary remnant pancreatic cancer or a metastatic cancer from other organs. A total remnant pancreatectomy was performed. The surgical duration was 229 min, and total blood loss was 251 mL. Her postoperative course was uneventful and she was discharged on 14th postoperative day.

**Fig. 6:**
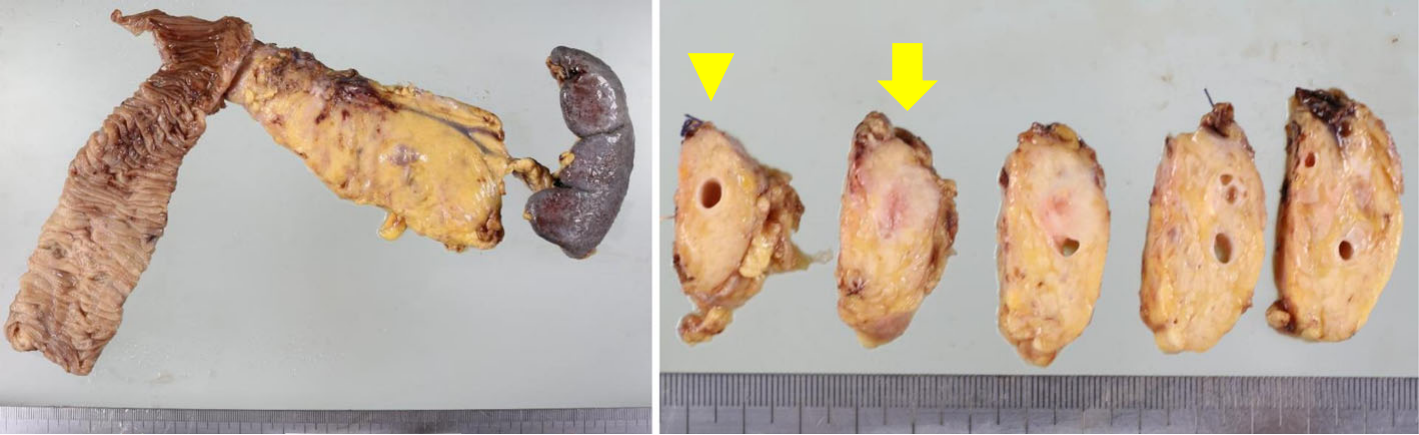
Macroscopic appearance of the resected specimens shows an irregular white tumor (arrow) which is located at the distal side of pancreaticojejunal anastomosis (arrow head)

**Fig. 7:**
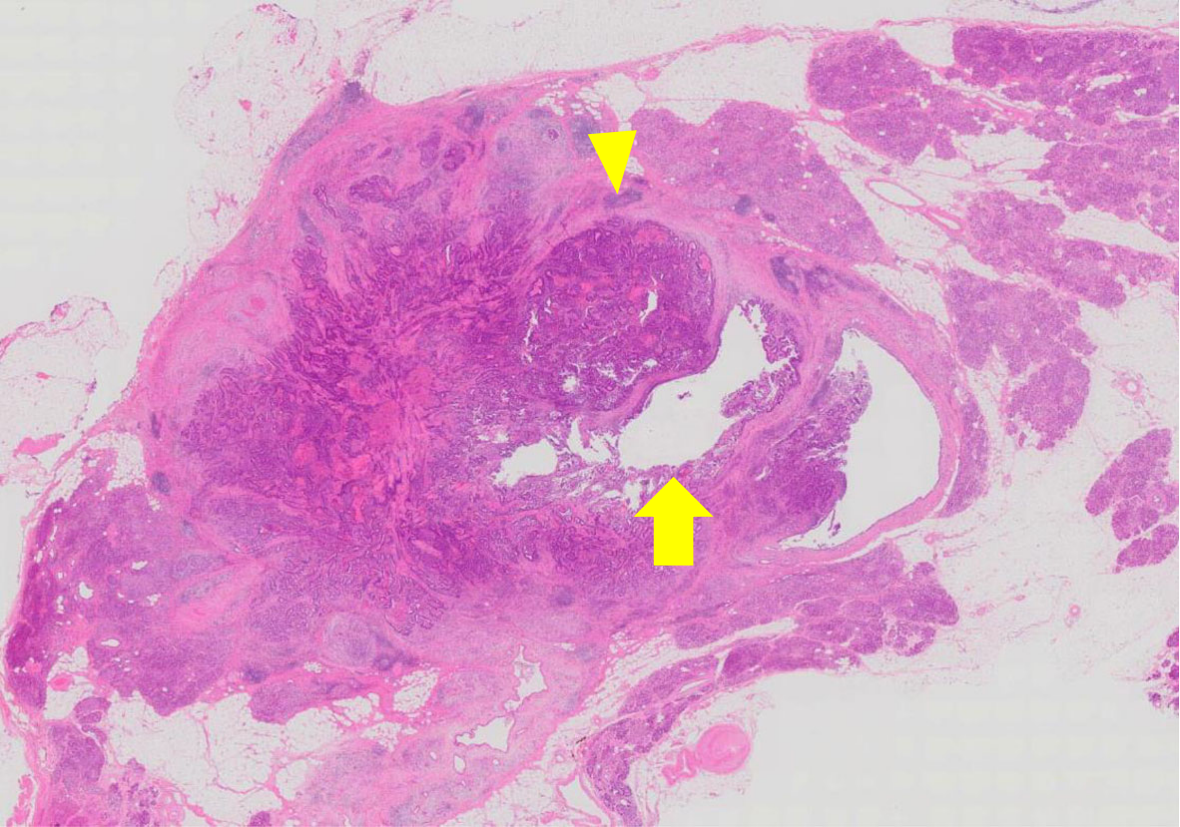
Microscopic findings showed adenocarcinoma cells (arrow head) invaded the main pancreatic duct epithelia (arrow) and the stroma. HE staining (× 10)

On gross examination of the resected specimen, recurrence in the pancreatic duct was observed at 2.5 cm from the pancreaticojejunal anastomosis (Fig. 6). There was neither invasion of lymphatics nor microvessels. There was no peritoneal dissemination or lymph node metastasis. The main tumor had infiltrated into the main duct and stroma (Fig. 7).

**Fig. 8:**
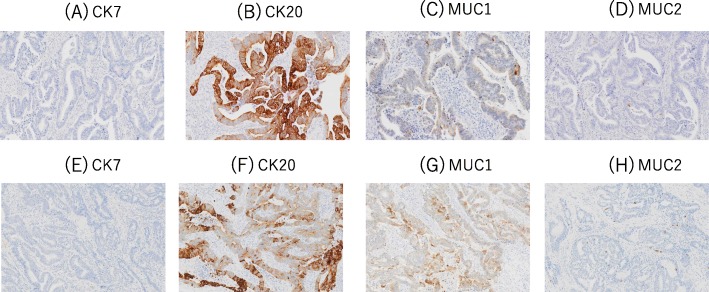
Immunohistochemical analysis revealed identical profiles between the original ampullary carcinoma (**a**–**d**) and the remnant tumor (**e**–**h**). Both sites were as follows: CK7-negative (**a**, **e**), CK20-positive (**b**, **f**), MUC1-positive (**c**, **g**), and MUC2-negative (**d***,* **h**)

The remnant tumor was morphologically similar to the original ampullary carcinoma. Immunohistochemical staining results were identical to those of the original specimen (Fig. 8). There were no other malignant findings nor atypical lesions in the remaining pancreatic duct epithelium on gross pathological examination. Ten months after the second surgery, there were no signs of recurrence.

## Discussion

The patterns of postoperative recurrence of ampullary carcinoma are locoregional recurrence and metastasis to the liver, peritoneum, lymph node, lung, or bone [1–3]. In many cases, there are simultaneous multiple metastases at recurrence; therefore, surgery (e.g., residual pancreatectomy) is seldom considered an option to for curative treatment. The risk factors for locoregional recurrence are the presence of pancreatic invasion and tumor size. By contrast, lymph node metastasis is the risk factor for distant recurrence [1]. There are few reports of ampullary carcinoma duplicated with pancreatic cancer.

Implantation of cholangiocarcinoma and lung cancer may occur. Transductal dissemination of pancreatic cancer has been observed in an animal model [4]. Intraductal dissemination of ampullary carcinoma was rare; however, some cases have been reported. Ban speculated that an identical acinar cell carcinoma suggested intraductal dissemination from the pancreatic tail to the pancreatic head [5]. Matsubara reported an adenocarcinoma that had disseminated to the pancreatic duct in a retrograde manner and recurred in the remnant pancreas after pancreaticoduodenectomy; the authors claimed that the case of intraductal dissemination would require confirmation of a non-invasive primary cancer of the pancreatic duct, with no invasion of lymphatic and venous duct, and multiple foci [6].

In our case, the pathological findings of the first operation showed no invasion in the pancreatic parenchyma, and the resection stump was negative for carcinoma. At the first operation, there was no obvious peritoneal dissemination or lymph node metastasis and no evidence suggesting lymphatic vessel invasion or vein invasion.

There are several pieces of evidence that suggests intraductal dissemination. First, the cancerous lesion was located at a site distant from the pancreatojejunal anastomosis. There were no malignant cells between the anastomosis and the tumor. Second, this tumor was identical to the original primary with respect to histology and immunohistochemistory. Third, there was a possibility of iatrogenic cancer implantation caused by the previous endoscopic biliary stenting prior to the first operation. The obstruction of the pancreatic duct or an iatrogenic procedure (ERCP) could have caused tumor cell implantation in the pancreatic duct epithelium in retrograde fashion.

It is unclear whether the tumor in this case was caused by tumor implantation or blood-borne metastasis. This tumor showed invasive growth to both the pancreatic parenchyma and to the epithelium of the main pancreatic duct. Therefore, the origin of the recurrent lesion remains unclear. Furthermore, because the growth rate of the ampullary carcinoma after implantation is unknown, the period from the first surgery to recurrence is not useful to determine the cause of recurrence.

## Conclusions

Intraductal dissemination of ampullary carcinoma is a cause of recurrence in the pancreatic remnant after pancreaticoduodenectomy in cases of obstruction of the pancreatic duct or an iatrogenic procedure preoperatively.

## Data Availability

Data sharing is applicable to this article.

## Abbreviations

CA19-9: Carbohydrate antigen 19-9
CEA: Carcinoembryonic antigen
CT: Computed tomography
ERCP: Endoscopic retrograde cholangiopancreatography
EUS: Endoscopic ultrasound
MRI: Magnetic resonance imaging
PET-CT: Positron emission tomography/computed tomography
SSPPD: Subtotal stomach preserving pancreatoduodenectomy
